# Gestational Age at Birth and ‘Body-Mind’ Health at 5 Years of Age: A Population Based Cohort Study

**DOI:** 10.1371/journal.pone.0151222

**Published:** 2016-03-14

**Authors:** Frances M. Cronin, Ricardo Segurado, Fionnuala M. McAuliffe, Cecily C. Kelleher, Richard E. Tremblay

**Affiliations:** 1 School of Public Health, Physiotherapy and Sports Science, University College Dublin, Dublin, Ireland; 2 UCD CSTAR, School of Public Health, Physiotherapy and Sports Science, University College Dublin, Dublin, Ireland; 3 UCD Obstetrics and Gynaecology, School of Medicine and Medical Science, University College Dublin, National Maternity Hospital, Dublin, Ireland; 4 Departments of Pediatrics and Psychology, University of Montreal, Montreal, Canada; The University of Queensland, AUSTRALIA

## Abstract

Numerous studies have identified the effects of prematurity on the neonate’s physical health, however few studies have explored the effects of prematurity on both the physical and mental health of the child as they develop. Secondary analysis of data from the Millennium Cohort Study, a longitudinal study of infants (n = 18 818, born 2000–2002 in the United Kingdom) was performed. Effects of gestational age at birth on health outcomes at 5 years were measured using parental rating of their children’s general health and severity of behavior problems. The association between parent’s general health ratings and behavior problem ratings was low: 86% of those reporting serious behavior problems (5% of the sample, n = 764) rated their child as being in excellent, very good, or good health. Still, a gradient of increasing risk of poorer outcome with decreasing gestational age was observed for a composite health measure (poor/fair health and/or serious behavior problems), suggesting an association with prematurity for this composite assessment of health status. The greatest contribution to the childhood composite health measure at 5 years was for children born at 32–36 weeks gestation: population attributable fractions for having poor outcomes was 3.4% (Bonferroni-adjusted 95% confidence interval 1.1%–6.2%), compared to 1% (0.2–2.3) for birth at less than 32 weeks. Results suggest that preterm children, by school entry, are not only at high risk of physical health problems, but also of behavioral health problems. The recognition of, and response to comprehensive health and well-being outcomes related to prematurity are important in order to correctly plan and deliver adequate paediatric health services and policies.

## Introduction

A relatively strong association between gestational age and health at age 5 years was recently described using the large population-based UK Millennium Cohort Study (MCS) [1]. An increase in health problems as gestational age decreased was reported as a”dose-response” effect of prematurity on children’s health at the age when most are starting compulsory education. However, while the Boyle *et al*., study [1] assessed numerous aspects of physical health (e.g. hospital admissions at 9 months and at 5 years, longstanding illnesses, asthma etc.) it did not specifically assess any dimension of mental health, except possibly through a measure of general health with parental ratings. This is not an unusual omission for population health surveys [2], but it is an important omission for a number of reasons: first, it has become clear that the origins of lifelong mental health problems can be assessed during early childhood [3–5]; second, mental ill-health has been identified as the single largest cause of disability in the UK, accounting for 22.8% of the total burden of disability and costing £105.2 billion each year [6]; third, childhood behavior problems such as emotional problems and conduct disorder are the most common and costliest of childhood health problems [7], with many physical and mental health issues being interrelated [8]; and finally, the need to integrate behavioral health services into mainstream paediatric primary care has recently been recognised as a matter of ‘national priority’ in some countries such as the US [9, 10], with the aim of diminishing the ‘body-mind’ separation (illness of the body v. illness of the mind) for children and promoting a model for optimal health care for the individual [11].

The aim of the present study was to use the same sample, and replicate the methodology described by Boyle *et al*., (2012) extending their findings by using both physical and mental health measures in order a) to assess to what extent at age 5 years the general health assessment by parental ratings encompasses severe behavior problems and b) describe the association between preterm birth and a combined assessment of physical and behavioral health.

## Materials and Methods

### The sample

The MCS began in 2002 and is a nationally representative prospective cohort study, based in the United Kingdom (England, Scotland, Wales) and Northern Ireland. Full details of the study have been reported previously [12], however a brief overview will be given here. Using the United Kingdom (UK)-based register of social payment of child benefit as eligibility criterion, all children born in England and Wales between 1 September 2000 and 31 August 2001, and in Scotland and Northern Ireland between 24 November 2000 and 11 January 2002 were included in the MCS if they were alive and living in the jurisdiction at the time of the first survey (MCS1). This resulted in a UK-wide longitudinal study of 18818 infants. Data were collected from parents of these children by trained researchers by means of a computer-assisted personal, face-to-face interview. The first survey interviewed parents when the proband child was approximately 9 months, the second survey (MCS2) took place when the child was 3 years old, and the third (MCS3) when the child was 5. Due to time constraints during the first sweep, health questions surrounding mother and baby health during pregnancy, birth and delivery were minimal. However, mothers were asked for their written consent to link their responses with birth registration and hospital records and these data were incorporated into the sweep database (matching took place for 99% of babies) [13].

The MCS sample design included geographical clustering (at an electoral ward level) and disproportionate stratification (using 9 different strata with all UK countries each having two strata: advantaged and disadvantaged and one additional strata for ethnic minority for England). This sampling design ensured adequate representation of ethnic minorities, areas of high child poverty and residents of the smaller countries. In order to replicate and extend from the health outcomes described by Boyle *et al*., [1] their baseline measures, categorical estimators, specific selection strategies and presentation methods were replicated: only cases where the natural birth mother was the respondent were used; exclusions were made for children with missing gestational age, those born after 42 weeks gestation, and those whose birth weight and gestational age were deemed improbable (based on Bonellie’s centile charts of birth weight for gestational age [14]). This resulted in total exclusions of n = 1198.

As this research involved secondary analysis of the MCS, ethical approval was not required.

### Gestational age

Gestational categories were calculated based on guidelines set down by the European Foundation for the Care of Newborn Infants [15] and divided as follows: preterm (<31 weeks), moderate preterm (32–33 weeks), late preterm (34–36 weeks), early term (37–38 weeks) and full term (39–41 weeks). While Boyle *et al*., (2012) used these standard categories to examine health outcome, they constructed an additional category—‘mature’ preterm (32–36 weeks) to explore specifically the mid-range of preterm birth in comparison to the extreme preterm and/or early/full term births [1]. For the purposes of comparison, this additional category was also constructed and examined in the present study.

### Outcome measures

General health at age 5 years was assessed by asking parents to rate their children’s general health as ‘excellent’, ‘very good’, ‘good’, ‘poor’, or ‘fair’. This question is part of the widely used and well validated ‘Child Health Measure Questionnaire Parent Form’ [16] that has been cross-culturally adapted and validated in a 32-country (n = 6,644) study as part of the Paediatric Rheumatology International Trials Organisation [17, 18].

Behavior problems were assessed using the parent-completed Strengths and Difficulties Questionnaire (SDQ) [19]–a well validated behavioral problems screening tool. A ‘Total Difficulties’ score of 17 or more across the population of children of all ages is regarded as ‘abnormal’ and is considered of use to screen for child psychiatric disorders in a community sample [19, 20].

### Statistical methods

The sampling design of the MCS was such that methods employed for simple random sampling or independence of observations could not be used as this would lead to an underestimation of standard errors and subsequent invalid significance tests. All analyses were therefore completed using Complex Sampling Plans (CSPLAN; SPSS Statistics 20) commands as outlined by Jones and Ketende [21]. Furthermore, all reported percentages and means were weighted for appropriate non-response and attrition weights appropriate for each survey [22].

Potentially confounding variables were controlled for, including child’s sex, child age at interview, ethnic group (White British or not), length of time breastfed (ever and longer than 4 months duration), mother’s age at time of birth, mother’s relationship status (single mother or not), mother’s education (degree level or not), if mother was employed in managerial/professional capacity or not, if mother was long-term unemployed, mother’s use of alcohol and/or tobacco during pregnancy.

Binary variables were created for the child’s SDQ Total Difficulties score being 17 or higher and also for parental rating of general health as ‘poor’ or ‘fair’. Results were combined to create a binary variable of ‘composite health’ identifying those who were rated as having fair or poor health and/or given a rating of 17 or higher on the SDQ Total Difficulties score at age 5.

Logistic regression was used to investigate the associations of adverse health outcome with gestational category, and unadjusted and fully adjusted models were run for each exposure-outcome group. The full term group was used as the comparison for the gestational categories. Multiple births (twins and triplets) were included for comparison purposes in the descriptive overview of the MCS sample, but were excluded from the regression analyses as multiple births are more likely to be PT—or induced PT [23].

In keeping with Boyle *et al*., (2012), population attributable fractions and associated 95% Confidence Intervals (CI) were calculated using the Bonferroni inequality method as described by Natarajan [24], which allows for the weighting, stratification and clustering that is associated with SPSS CSPlans.

## Results

### The sample

The MCS1 collected data on 18818 children. For this analysis, exclusions described by Boyle *et al*., (2012) were made (including children with no information on gestational age, those born later than 42 weeks, those whose birth weight and gestational age were deemed improbable, and where the natural mother was not the respondent). This resulted in a final sample for MCS1 of 17620. The MCS3 took place when the proband child was 5 years old and provided parental ratings of general health (n = 14087) and a ‘Total Difficulties’ score on the SDQ (n = 13427) [22].

Table 1 describes the maternal characteristics at MCS1, when the children were age 9 months. Compared to mothers of children born at 33 weeks and later, mothers of children born before 32 weeks were, on average, the youngest mothers, were least likely to hold a degree, and most likely to have no formal qualification. A greater proportion of them smoked while pregnant, and following the birth, a higher percentage did not breastfeed (with a lower percentage breastfeeding for longer than 4 months).

**Table 1:** Characteristics of mothers of children included in the analysis. Values are numbers (percentages) unless otherwise stated.

	Gestation at birth (weeks)					
Mother’s characteristics	<32 (n = 206)	32–33 (n = 184)	34–36 (n = 1058)	37–38 (n = 3607)	37–41 (n = 12565)	P values comparing gestational age groups^a^
Mean (95% CI) age at time of child’s birth (yrs)	28.4 (27.3–29.5)	29.7 (28.5–30.9)	28.9 (28.4–29.5)	29.2 (28.9–29.5)	28.7 (28.5–29.0)	0.001
Single mother	41 (18.5)	26 (9.4)	208 (16.4)	603 (14.0)	2104 (14.1)	0.065
Socioeconomic status:						0.414
Managerial and professional	50 (24.6)	45 (27.9)	275 (30.6)	922 (29.0)	3356 (30.5)	
Long term unemployed	21 (7.5)	21 (8.8)	118 (8.6)	417 (8.6)	1422 (8.2)	
Education:						<0.001
Degree	59 (29.8)	50 (31.3)	297 (30.0)	994 (30.2)	3752 (33.7)	
No qualifications	47 (21.6)	28 (11.3)	184 (14.1)	626 (14.4)	1948 (12.3)	
Moderate/heavy alcohol use during pregnancy^b^	12 (7.0)	12 (6.8)	61 (6.9)	237 (6.7)	885 (7.3)	0.917
Smoked during pregnancy	59 (29.4)	46 (17.9)	286 (25.0)	824 (21.3)	2734 (20.2)	0.003
Duration of breastfeeding						<0.001
Never	62 (28.2)	52 (27.9)	415 (35.1)	1327 (33.4)	4220 (29.5)	
≥ 4 months	39 (19.2)	36 (23.0)	222 (23.6)	951 (28.9)	3883 (34.6)	

Means and percentages are weighted with sampling and non-response weights

^a^χ^2^ tests for categorical variables and F tests for continuous variables; calculated for weighted, clustered data

^b^≥ 3 units/week or ≥ 3 units/occasion

The characteristics of the children involved in the MCS1 are reported in Table 2. The average birth weight of the children increased as gestational age increased. A greater proportion of those born before 32 weeks were from groups other than White British. Being of multiple birth and/or first born increased the likelihood of being born before 34 weeks. Those born before 32 weeks gestation had the longest average stay in neonatal care. Children significantly differed in their age at the time of the 5 year interview, but this was by less than 0.1 years on average.

**Table 2:** Characteristics of children from the Millennium Cohort Study included in this analysis. Values are numbers (percentages) unless otherwise stated.

			Gestation at birth (weeks)			
Child’s characteristics	<32 (n = 206)	32–33 (n = 184)	34–36 (n = 1058)	37–38 (n = 3607)	37–41 (n = 12565)	P values comparing gestational age groups^a^
Mean (95% CI) birth weight (kg)	1.3 (1.2–1.3)	1.9 (1.9–2.1)	2.6 (2.5–2.6)	3.1 (3.1–3.2)	3.5 (3.5–3.5)	<0.001
Male sex	123 (57.9)	96 (57.3)	566 (53.7)	1882 (51.9)	6370 (50.6)	0.081
Ethnic group:						<0.001
White British	156 (79.5)	153 (88.4)	880 (88.1)	2919 (85.6)	10516 (87.9)	
Other^b^	50 (20.5)	30 (11.6)	178 (11.9)	683 (14.4)	2029 (12.1)	
Multiple birth	39 (20.3)	43 (28.3)	178 (17.1)	178 (5.2)	59 (0.4)	<0.001
First born	103 (51.3)	93 (52.9)	497 (47.9)	1342 (37.6)	5369 (42.5)	<0.001
Mean (95% CI) length of neonatal hospital stay (wks)	9.1 (8.0–10.2)	3.3 (3.0–3.6)	1.4 (1.3–1.6)	0.5 (0.5–0.6)	0.4 (0.4–0.4)	<0.001
Mean (95% CI) age at 5 year interview (yrs)	5.2 (5.1–5.2)	5.2 (5.1–5.3)	5.2 (5.2–5.2)	5.2 (5.2–5.2)	5.2 (5.2–5.2)	0.020

Means and percentages are weighted with sampling and non-response weights

^a^χ^2^ tests for categorical variables and F tests for continuous variables; calculated for weighted, clustered data

^b^Mixed, Indian, black Caribbean/black African, Bangladeshi/Pakistani, other

#### Attrition

The response rate at MCS3 (age 5) was 79% of eligible MCS families. In an analysis only weighted for MCS1 (baseline) non-response and the cluster sampling design, it was found there were significant socio-demographic differences between the mothers who took part in the age 5 sweep and those who did not (Table 3). Mothers who took part were—at the time of the child’s birth—more likely to have been older and working in managerial/professional roles and more likely to have breastfed their child for longer than 4 months. Fewer single mothers, mothers who had smoked during pregnancy and mothers who had never breastfed participated in MCS3.

**Table 3:** Univariate analysis of predictors for lost to MCS3 (age 5) follow-up of children enrolled at MCS1 (9 months). Weighted for complex sampling and non-response at baseline. n(%) and OR(95% CI) unless otherwise stated.

Mother’s characteristics	Followed up at age 5		Lost to follow-up		OR (95% CI)	Sig. (2-tailed)
	n	n(%)	n	n(%)		
Mother’s age at time of child’s birth (yrs) M(SE)	13972	29.14 (0.12)	3646	27.61 (0.17)	*1.53 (1.26–1.80)	<0.001
Single mother	13973	2125 (12.5)	3647	857 (21.5)	0.52 (0.47–0.58)	<0.001
Managerial/professional role	13973	3983 (32.3)	3647	665 (20.9)	1.81 (1.61–2.03)	<0.001
Long term unemployed	13973	1415 (7.1)	3647	584 (13.2)	0.51 (0.44–0.658)	<0.001
Degree	11599	4398 (40.3)	2645	754 (30.3)	1.55 (1.40–1.72)	<0.001
No qualifications	13595	1982 (11.1)	3510	851 (20.6)	0.48 (0.43–0.54)	<0.001
Moderate/heavy alcohol use during pregnancy^a^	13973	958 (7.1)	3647	249 (7.2)	0.98 (0.83–1.16)	0.822
Smoked during pregnancy	13973	3030 (19.8)	3647	919 (24.8)	0.75 (0.68–0.83)	<0.001
Never breastfed	13973	4328 (27.0)	3646	1524 (39.4)	0.57 (0.52–0.62)	<0.001
Breastfed for ≥ 4 months	13973	4295 (34.1)	3647	836 (25.5)	1.51 (1.36–1.68)	<0.001
**Child characteristics**						
Gestation time (days) M(SE)	13973	274.65 0.18	3647	273.96 (0.28)	*0.69 (0.04–1.34)	0.038
Gestation (weeks) M(SE)	13973	39.24 (0.03)	3647	39.13 (0.04)	*0.10 (0.01–0.19)	0.038
Birthweight (gs) M(SE)	13973	3336.33 (6.27)	3647	3300.71 (12.59)	*35.62 (8.10–63.14)	0.011
Length neonatal stay (wks) M(SE)	13973	0.60 (0.02)	3647	0.64 (0.03)	*0.04 (0.03–0.11)	0.242
Male sex	13973	7119 (51.0)	3647	1915 (52.0)	0.96 (0.88–1.04)	0.331
White British	13951	11780 (88.4)	3643	2844 (82.9)	0.63 (0.57–0.71)	<0.001
Gestational category	13973		3647			0.103
Very preterm		153 (1.1)		53 (1.4)		
Moderate preterm		146 (1.2)		38 (0.8)		
Late preterm		830 (6.0)		228 (6.4)		
Early term		2813 (20.1)		794 (22.0)		
Full term		10031 (71.6)		2534 (69.4)		

*Mean difference (95% CI)

^a^≥ 3 units/week or ≥ 3 units/occasion

Similarly, there were significant differences between the cohort of children assessed at age 5, and those children who were lost to follow-up. Children lost to follow-up were slightly younger in terms of gestation, with participants more likely to be full term with a smaller proportion of them in the ‘very preterm’ category.

### Fair or poor health

At age 5, fair or poor health was reported for 4.4% (n = 614) of the 14087 for which parents had rated the health of the child (see Table 4a). In unadjusted analysis, the highest odds ratio (OR) was for the moderate preterm (32–33 weeks) group (OR: 3.2 [95%CI: 1.8 to 5.6]), with the OR reducing as gestational age increased: <32 weeks OR: 2.0 [1.0 to 3.7]; 34–36 weeks OR: 1.9 [1.2 to 2.4]; 37–38 weeks OR: 1.5 [1.2 to 1.9].

**Table 4:** (a) Odds ratios of parent rating child’s health as fair or poor compared with excellent, very good, or good at 5 years (b) Odds ratios of parent rating child’s behavior with Total Difficulty score ≥17 compared with a Total Difficulty score <17 at 5 years.

	Gestation at birth (weeks)				
**a) Parental rating of Fair/Poor Health at 5 years**	**<32 (n = 158)**	**32–33 (n = 160)**	**34–36 (n = 846)**	**37–38 (n = 2845)**	**37–41 (n = 10078)**
N (%)	14 (6.4)	16 (10.1)	46 (5.5)	154 (5.1)	384 (3.4)
Unadjusted odds ratio (95% CI)	2.0 (1.0 to 3.7)	3.2 (1.8 to 5.6)	1.7 (1.2 to 2.4)	1.5 (1.2 to 1.9)	1
Adjusted^a^ odds	1.8 (.9 to 3.4)	3.5 (1.9 to 6.7)	1.6 (1.1 to 2.3)	1.5 (1.1 to 1.8)	1
Adjusted^a^ population attributable fraction—% (95%)	0.8 (-0.2 to 2.6)	2.1 (0.6 to 4.6)	3.1 (0.2 to 7.2)	8.1 (2.1 to 14.9)	
		**32–36 (n = 1006)**			
Adjusted^a^ population attributable fraction—% (95%)		5.2 (1.7 to 9.5)			
**b) SDQ**^b^ **Total Difficulty score ≥ 17 at 5 years**					
N (%)	19 (12.5)	9 (5.5)	47 (6.8)	169 (5.9)	520 (5.3)
Unadjusted odds ratio (95% CI)	2.6 (1.5 to 4.4)	1.1 (0.5 to 2.3)	1.3 (1.0 to 1.8)	1.1 (0.9 to 1.4)	1
Adjusted^a^ odds	2.3 (1.3 to 3.9)	1.2 (0.5 to 2.8)	1.2 (0.9 to 1.7)	1.1 (0.8 to 1.4)	1
Adjusted^a^ population attributable fraction—% (95%)	1.4 (0.3 to 3.4)	0.2 (-0.6 to 1.8)	1.3 (-1.0 to 4.5)	1.9 (-4.0 to 8.5)	
		**32–36 (n = 1006)**			
Adjusted^a^ population attributable fraction—% (95%)		1.5 (-1.1 to 4.9)			

All percentages and odds ratios are weighted with sampling and non-response weights

^a^Adjusted for child’s age at interview, sex, ethnicity, maternal age at birth, marital status, education, occupation, duration of breastfeeding, maternal smoking and alcohol intake during pregnancy

^b^Strengths & Difficulties Questionnaire [19]

Adjusted analysis again showed the highest risk to be for the 32–33 week category in comparison to full term (OR 3.5 [1.9 to 6.7]), with the OR reducing as gestational age increased: the OR for the extreme preterm (<32 weeks) approached significance at 1.8 [0.9 to 3.4], for those born between 34–36 weeks OR 1.6 [1.1 to 2.3], and for early term (37–38 weeks) OR: 1.5 [1.1 to 1.8]. The adjusted population attributable fraction for the additional category of the ‘mature’ preterm (32–36 weeks) group was 5.2 [1.7 to 9.5].

### Behavior problems

At age 5, 5.6% (n = 764) of the children had SDQ Total Difficulties scores of 17 or higher (see Table 4b). The greatest proportion of children with problem behavior was in the <32 weeks category, with OR for having an SDQ Total Difficulties score of ≥17 significant only for this group (unadjusted OR 2.6; [1.5 to 4.4]; adjusted OR 2.3 [1.3 to 3.9]).

### Association between general health assessment and behavior problems

There was an association between parent’s general health ratings and behavior problem ratings (p<0.001). Only 21% (n = 107) of the 613 children rated as having poor or fair physical health (4.4%) also had behavior problems (scores of 17+ on the SDQ).

Of the 764 parents who rated their child as having serious behavior problems (5.6%) only 14.3% (n = 107) rated their child as also being in poor or fair health. Despite an approximately 3-fold higher risk of behavior problems among children with poor or fair physical health ratings, this suggests that for many parents, a child with serious behavior problems is not considered to be suffering from poor health (n = 657).

Further analysis showed that of the 14.3% of children who were rated as having both serious behavior problems and poor health, 77.3% (86/107) had what the parents defined as a ‘long-term illness’ indicating a concurrent physical health problem which may be driving the poor/fair health rating. Conversely, of those with a longstanding illness, only 11% (282/2618) had ratings that indicated behavior problems.

### Association between the composite health measure and gestational age

A gradient of increasing risk of poorer outcome with decreasing gestational age was observed for the composite health measure (poor/fair health and behavior problems), suggesting a trend effect of prematurity for this composite assessment of health status (see Table 5).

**Table 5:** Odds ratios of poor composite health (SDQ^b^ Total Difficulties score ≥17 and/or parent rating child's health as fair or poor) compared with good composite health (SDQ score <17 and/or parent rating child's health as excellent, very good, or good) at 5 years.

			Gestation at birth (weeks)		
Adverse composite health score at 5 years	<32 (n = 153)	32–33 (n = 146)	34–36 (n = 830)	37–38 (n = 2814)	37–41 (n = 10032)
N (%)	27 (15.7)	23 (14.4)	86 (11.2)	296 (9.8)	839 (7.8)
Unadjusted odds ratio (95% CI)	2.2 (1.4 to 3.5)	2.0 (1.2 to 3.1)	1.5 (1.2 to 1.9)	1.3 (1.1 to 1.5)	1
Adjusted^a^ odds	2.0 (1.3 to 3.2)	2.2 (1.3 to 3.8)	1.4 (1.1 to 1.8)	1.2 (1.0 to 1.5)	1
Adjusted^a^ population attributable fraction—% (95%)	1.0 (0.2 to 2.3)	1.1 (0.2 to 2.4)	2.3 (0.3 to 4.8)	4.4 (-0.2 to 9.3)	
		3.4 (1.1 to 6.2)			

All percentages and odds ratios are weighted with sampling and non-response weights

^a^Adjusted for child’s age at interview, sex, and ethnicity, maternal age at birth, marital status, education, occupation, duration of breastfeeding, maternal smoking and alcohol intake during pregnancy

^b^Strengths & Difficulties Questionnaire [19]

The composite assessment of health status identified 9.1% of the population (n = 1271) as having health difficulties, with a clear trend effect of prematurity on the percentage found in each gestational categories (7.8% in full term, increasing to 15.7% in extreme preterm). In unadjusted analysis, a similar effect was seen with higher OR in preterm OR: 2.2 [1.4 to 3.5] which reduced as gestational age increased: 32–33 weeks OR: 2.0 [1.2 to 3.1]; 34–36 weeks OR: 1.5 [1.2 to 1.9], 37–38 weeks OR: 1.3 [1.07 to 1.54]. Adjusted analysis showed a slightly increased risk for the 32–33 category in comparison to the extreme preterm category (<32 weeks): OR: 2.2 [1.3 to 3.8] and OR: 2.0 [1.3 to 3.2] respectively.

Population attributable fractions for having poor outcomes in the composite health score at 5 years were 1.0% (Bonferroni-adjusted 95% CI 0.2% to 2.3%) for birth before 32 weeks, compared with 4.4% (n/s) for children born at 37–38 weeks. There was a trend effect for 32–33 weeks and 34–36 weeks, with results of 1.1 [0.2 to 2.4] and 2.3 [0.3 to 4.8] respectively.

## Discussion

The present study had two aims: firstly, to explore to what extent parents consider serious behavioral problems when rating their child’s general health at age 5 years, and second, to describe the association between gestational age at birth and a composite health assessment which includes both general health ratings and serious behavioral problems.

With reference to the first aim of the study, the results clearly show that most parents do not consider serious behavioral problems when rating their child’s general health at age 5 years: while a clear gradient of increasing risk of poorer outcome with decreasing age for both general health rating and behavior problems rating suggests a trend effect of prematurity for each outcome, 86% of the parents who reported that their child had behavior problems of clinically relevant levels rated the same child as being in excellent, very good, or good health. Of the parents who reported their child as having both a serious behavior problem and being of poor/fair health, 77.3% (86/107) of their children had a ‘long term’ illness, indicating a concurrent physical health problem. Conversely, of those with a longstanding illness, only 11% (282/2618) had behavior ratings that might indicate a behavior problem clinical diagnosis.

These results suggest that parents (and most likely the general public) do not consider behavioral problems of young children as ‘health’ problems. Despite the WHO defining health as a ‘state of complete physical, mental and social well-being and not merely the absence of disease or infirmity’ [25], a distinction between mental and physical illness persists in both lay and medical circles, and continues in scientific publications [11]. Correcting this tendency could have long term beneficial impacts since behavioral problems during early childhood have been shown to be important risk factors for adult mental health problems—the largest and most costly cause of disability in the UK [6, 7].

With reference to the second aim of the study, finding the association between gestational age at birth and health at age 5 years, a gradient of increasing risk of poorer outcome with decreasing gestational age was observed for both the general health rating and the behavior problems rating, suggesting a trend effect of prematurity for each outcome.

While our findings confirm the results for general health rating reported by Boyle *et al*., [1] with the same magnitude and direction of the effect of gestational age and a trend effect of having poor or fair health which increased as gestational age reduced, our results show the incidence of serious behavior problems also increased with decreasing gestational age.

However, serious behavior problems were significantly associated only with extreme preterm birth (<32 weeks). These results—using one of the largest and most recent population surveys in the UK—are in keeping with a number of previous studies reporting that at age 5, children born preterm exhibit higher levels of behavior problems [26] and/or psychological problems [26] in comparison with children born at full term, with an increased risk of problem behavior specifically for those born before 32 weeks [27].

The present study shows that a composite health measure (a combination of the parent’s health ratings of poor/fair and serious behavior problem ratings) provides a more powerful and accurate measure of children’ health status at age 5 years. While maintaining the gradient of increasing risk of poorer outcome with decreasing gestational age, the addition of behavior problem cases to the general health problem cases makes a substantially greater contribution to the <32 weeks gestational category (+ 9.3%) compared to the other categories (range 4.3% to 5.7%). The mothers from this gestational age category tended to be younger, less educated and more likely to smoke during pregnancy. This is a maternal profile that has been associated with behavior problems of the mothers during their own childhood [28]. Thus, the association between gestational age and physical/behavior problems of the children at age 5 years may be due to a mixture of genetic, epigenetic and environmental risks factors. To unravel these complex mechanisms we will need studies that control for genetic effects and monitor both epigenetic and environmental contributions from pregnancy onwards [29].

From an adjusted population attributable fraction perspective it remains clear that the gestational categories between 32 and 36 weeks still provide a substantial number of children with adverse health problems (physical and/or mental) suggesting that preventive interventions with some of these families could significantly reduce physical and behavioral problems by school entry. These interventions might specifically target pregnant women with risk factors associated with low GA deliveries such as low or poor education, tobacco or alcohol use while pregnant, poverty and lone-motherhood [30]. The preventive interventions should also target low GA children with behavioral and cognitive interventions that have been reported to have long term impacts on children with behavioral problems [31, 32].

The findings from the present study are important as the complications in health terms that are associated with prematurity present not only a large burden to the individual and family, but also a ‘substantial and increasing’ impact on a country’s health resources [33]. With the growing rates of preterm births together with increased survival rates comes an increase in pathology and risk of long-term poor physical and mental health conditions that are part of a sequela of being born prematurely [34]. In order to plan early preventive interventions and allocate necessary resources that will deliver the optimum outcome, it is important to have accurate estimations of physical and mental health outcomes. Preventive interventions are particularly important in the children’s younger years when they have the lowest risk of stigma [9], and are considered as having the highest likelihood of long term effects and provide the best return on investment [35].

An important strength of the present study is the magnitude of the sample size. The MCS is one of the largest population-based longitudinal studies to provide both neonatal information together with physical and behavioral health outcomes at school entry age. This allows a comparison of a variety of gestational age groups in relation to physical and behavioral health outcomes at an age when serious problems can be relatively well assessed. Despite this strength, some limitations need to be acknowledged. First, as can be observed from our analyses, the number of subjects in the lowest gestational age categories is still relatively small. Second, despite weighting for non-response and sampling at baseline, there were significant differences between responders and non-responders at age 5. To correct for putative biases we used weights for longitudinal non-responses, and fit adjusted logistic regression models using maximum likelihood, which are expected to perform well under ignorable missing data patterns [22]. Finally, it is important to note that the SDQ assessment gives a good estimate of the prevalence of serious behavior problems at age 5 years with reference to the gestational age groups, but a more precise estimate of the long-term impact of gestational age—including any potential confounding of maternal mental health status—will require further assessments during childhood, adolescence and adulthood [36, 37].

In summary, lifelong mental health issues are currently the biggest contributors to health services costs [6, 7]. Precursors of these conditions—problem behavior in childhood—can be identified in early childhood [4, 38]. Preterm children are vulnerable not only to physical health problems, but also to adverse behavioral outcomes [26, 27]. The increase in both preterm delivery and preterm birth survival has had an impact on the number of children presenting with adverse behavioral problems [34]. While studies into the effects of early interventions for preterm children continue to provide mixed results [39–41], there is evidence that intensive long term interventions with at-risk pregnant women can have some long term effects on the physical and mental health of their children [31, 32]. Similarly, intensive interventions at the start of elementary school with preschoolers that had behavior problems have shown long term impacts [42, 43].

We suggest that a new generation of prevention studies should target pregnant women at very high risk of giving birth early (e.g. low or poor education, tobacco or alcohol use while pregnant, poverty and lone-motherhood). These interventions should start during pregnancy and last throughout early childhood with an emphasis on preventing both physical and mental health problems. They should give support to the parents as well as the child in both the home, preschool and school environment. This preventive approach would complement the policy of inclusion of behavioral and physical health measures for use in paediatric primary care that is currently being adopted as a ‘national priority’ in some countries [9]. A rigorous long-term evaluation framework would be needed and would require substantial investment for implementation and evaluation.

In conclusion, results of the present study show that assessments of the impact of gestational age cannot be limited to physical health only. Behavioral health is an important aspect of the health of an individual—particularly during early childhood. Continuing to view prematurity as having only physical health outcomes leads to a neglect of some of its most costly long term outcomes—mental health problems. This in turn, may lead to the failure to provide timely, effective, meaningful preventive interventions. Further studies of gestational age impact need to consider other forms of long term unwanted outcomes associated with early childhood health problems such as school failure and failure to enter the job market. The recognition of, and response to comprehensive health and well-being issues related to prematurity are needed in order to correctly plan and deliver adequate paediatric health services and policies.
